# Uncoupling the Threading and Unfoldase Actions of *Plasmodium* HSP101 Reveals Differences in Export between Soluble and Insoluble Proteins

**DOI:** 10.1128/mBio.01106-19

**Published:** 2019-06-04

**Authors:** Kathryn M. Matthews, Ming Kalanon, Tania F. de Koning-Ward

**Affiliations:** a School of Medicine, Deakin University, Waurn Ponds, Victoria, Australia; NIAID/NIH

**Keywords:** AAA^+^-ATPase, HSP101, PTEX, *Plasmodium*, genetic engineering, protein trafficking, unfoldase

## Abstract

*Plasmodium* parasites must export proteins into their erythrocytic host to survive. Exported proteins must cross the parasite plasma membrane (PPM) and the parasitophorous vacuolar membrane (PVM) encasing the parasite to access the host cell. Crossing the PVM requires protein unfolding and passage through a translocon, the *Plasmodium* translocon of exported proteins (PTEX). In this study, we provide the first direct evidence that heat shock protein 101 (HSP101), a core component of PTEX, unfolds proteins for translocation across the PVM by creating transgenic *Plasmodium* parasites in which the unfoldase and translocation functions of HSP101 have become uncoupled. Strikingly, while these parasites could export native proteins, they were unable to translocate soluble, tightly folded reporter proteins bearing the *Plasmodium* export element (PEXEL) across the PVM into host erythrocytes under the same conditions. In contrast, an identical PEXEL reporter protein but harboring a transmembrane domain could be exported, suggesting that a prior unfolding step occurs at the PPM. Together, these results demonstrate that the export of parasite proteins is dependent on how these proteins are presented to the secretory pathway before they reach PTEX as well as their folded status. Accordingly, only tightly folded soluble proteins secreted into the vacuolar space and not proteins containing transmembrane domains or the majority of erythrocyte-stage exported proteins have an absolute requirement for the full unfoldase activity of HSP101 to be exported.

## Abstract (precis)

The *Plasmodium* parasites that cause malaria export hundreds of proteins into their host red blood cell (RBC). These exported proteins drastically alter the structural and functional properties of the RBC and play critical roles in parasite virulence and survival. To access the RBC cytoplasm, parasite proteins must pass through the *Plasmodium* translocon of exported proteins (PTEX) located at the membrane interfacing the parasite and host cell. Our data provide evidence that HSP101, a component of PTEX, serves to unfold protein cargo requiring translocation. We also reveal that addition of a transmembrane domain to soluble cargo influences its ability to be translocated by parasites in which the HSP101 motor and unfolding activities have become uncoupled. Therefore, we propose that proteins with transmembrane domains use an alternative unfolding pathway prior to PTEX to facilitate export.

## INTRODUCTION

*Plasmodium* parasites invade erythrocytes to induce the symptoms and pathologies associated with malaria. After invasion of an erythrocyte, the *Plasmodium* parasite develops within a parasitophorous vacuole (PV), drastically modifying the host cell, both structurally and biochemically (1, 2). This modification process requires hundreds of parasite proteins to be trafficked beyond the parasitophorous vacuolar membrane (PVM) encasing the parasite into the host erythrocyte (3–9). These exported proteins contribute to virulence and survival of the intracellular parasite (1, 10, 11).

There are two types of exported parasite proteins: (i) those that contain a 5-amino-acid motif termed the *Plasmodium* export element (PEXEL) (4) or host-targeting (HT) (3) motif downstream of a recessed amino-terminal signal sequence that directs entry into the endoplasmic reticulum (ER) and (ii) PEXEL-negative exported proteins (PNEPs) that do not possess a defined export motif. Instead, this smaller repertoire of exported proteins requires its N terminus in conjunction with a recessed transmembrane domain (TMD) to direct export (9, 12, 13). There are also examples of PNEPS that contain a classical N-terminal signal peptide with no predicted TMD (9).

Export of PEXEL proteins begins with translocation, or cotranslocation, into the ER via the Sec61 translocon (in either a Sec22-dependent or -independent manner) (14). The PEXEL motif is cleaved by plasmepsin V (15, 16), and the new N terminus is acetylated (17, 18). The mature PEXEL cargo is then loaded into secretory vesicles. At the parasite plasma membrane (PPM), soluble proteins lacking TMDs are released into the PV (19). However, the trafficking of PEXEL proteins containing TMDs (PEXEL-TMD proteins) is less well understood. Whether these PEXEL-TMD proteins are trafficked as soluble chaperoned complexes or, as has been shown for PNEPs (13, 20), inserted into the ER membrane during trafficking, has not been investigated. If PEXEL-TMD proteins are indeed inserted into the ER membrane, secretory vesicle trafficking would most likely incorporate the exported proteins into the PPM in a manner similar to that of the PNEPs. How these proteins are subsequently extracted out of the PPM to traffic further is unknown.

Regardless of how the cargo enters the PV, the trafficking pathways for mature PEXEL and PNEP proteins converge at the PVM, where both types of exported protein classes are actively translocated across the PVM via the *Plasmodium* translocon of exported proteins (PTEX). For clarity, this paper distinguishes the process of secretion across the PPM as opposed to the process of export across the PVM. The PTEX machinery comprises five proteins (21): heat shock protein 101 (HSP101), PTEX150, EXP2, PTEX88, and TRX2. In particular, HSP101, EXP2, and PTEX150 are essential for protein export and parasite survival (22–27). Compromising these core PTEX components, either by inducible knockdown approaches or constitutive truncations, results in a dramatic blockage of protein export and an accumulation of both PEXEL and PNEP cargo at the parasite-host cell interface (24–27). In contrast, PTEX88 and TRX2 are not essential for parasite survival but nevertheless contribute to parasite cytoadherence, sequestration, and virulence (22, 23, 28, 29).

The structure of the core PTEX complex has recently been solved by cryo-electron microscopy (cryo-EM) (30). This structure corroborated that EXP2 is the PVM pore-forming component, in keeping with several studies that showed that EXP2 is the most resistant of the PTEX components to carbonate extraction (21, 31), the finding that recombinant EXP2 protein has pore-forming activity in whole blood (32), and that a peptide derived from the N-terminal region of EXP2 is hemolytic (33). The structure resolved only 20% of PTEX150 but was sufficient to show that it forms a seven-unit oligomer ring and confirmed previous findings that PTEX150 associates with both HSP101 and EXP2 (31) and is likely to have a structural role in the PTEX complex. Previous studies have postulated this structural role for PTEX150, including experiments demonstrating that truncation of the C terminus of PTEX150 leads to reduced complex stability (34). HSP101, the third core component of PTEX, was observed in the cryo-EM structure to sit directly above the PTEX150/EXP2 pore. HSP101 is a member of the type I HSP100 ClpB/AAA+-ATPases, which canonically form ring-shaped hexamers with a central channel. Related ClpB proteins are characterized by a unique N-terminal domain that binds substrate proteins, either directly or through adapter proteins, and two conserved AAA+ domains that bind and hydrolyze ATP (35). In addition, they contain a unique coiled-coil middle domain that regulates ATPase and unfoldase activities (36–38) and a short C-terminal domain, which is essential for hexamerization (39, 40). The cryo-EM structure of HSP101 confirms an architecture similar to those of the canonical HSP100/ClpB proteins. This is consistent with the proposed role of HSP101 as an unfoldase that utilizes ATP to provide the motor force to drive protein translocation through the PTEX pore. Other members of the HSP100 family have been shown to serve as molecular motors for the translocation of proteins across membranes, such as HSP93 for import of proteins into chloroplasts (41) as well as Cdc48 for protein import into Apicomplexan apicoplasts and retrotranslocation of TM proteins from the ER membrane (42, 43).

One important facet of protein translocation across the PVM is that the cargo must be unfolded to traverse this membrane (44). It has been proposed that HSP101 threads cargo directly into the PTEX channel via its ATPase activity and is required for unfolding cargo (21, 39). Most HSP100/ClpB family members engage with cochaperones to unfold proteins to disassemble and disaggregate proteins (35, 37, 45–47). Whether HSP101 works independently of a cochaperone remains to be tested.

To characterize the function of HSP101 in *Plasmodium*, we reengineered the *hsp101* locus of Plasmodium berghei so that a chimeric HSP101-mCherry protein is expressed and incorporated into the PTEX complex. Incorporation of the chimeric HSP101 renders PTEX only partially functional and impairs translocation of different types of cargo across the PVM. Tightly folded soluble PEXEL-bearing reporter proteins are blocked from translocating across the PVM, while native parasite proteins and PEXEL-bearing green fluorescence protein (GFP) reporter proteins containing TMDs appear to be efficiently exported. Our data provide the first direct evidence that HSP101 functions as the PTEX unfoldase and that the folded and solubility states of cargo determine how proteins are translocated across the PVM.

## RESULTS

### Expression of a HSP101 chimera in P. berghei.

**FIG 1:**
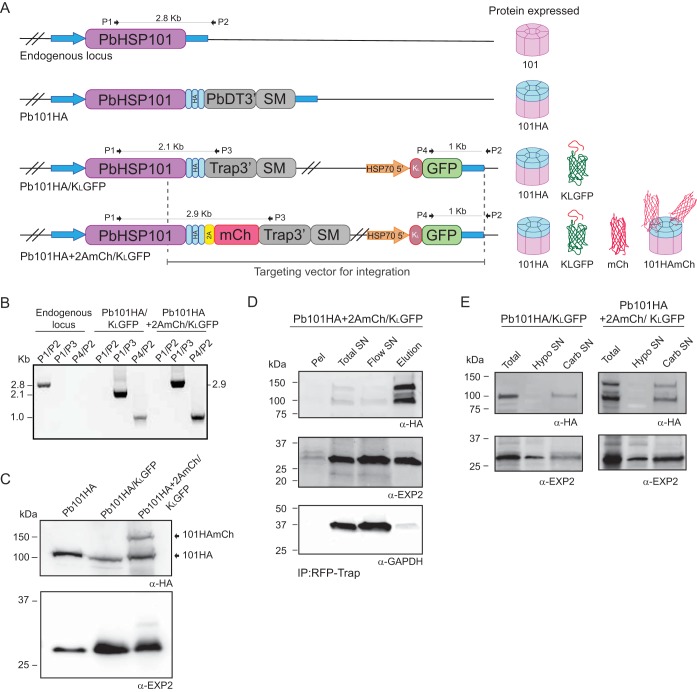
Generation of P. berghei transgenic parasites Pb101HA/K_L_GFP and Pb101HA+2AmCh/K_L_GFP. (A) Schematic representations of the endogenous P. berghei *hsp101* locus, Pb101HA, Pb101HA/K_L_GFP, and Pb101HA+2AmCh/K_L_GFP. Pb101HA is a previously generated parasite created by inserting a triple hemagglutinin tag (HA) at the C terminus of Hsp101 (22). Pb101HA/K_L_GFP was created by a double-crossover recombination leading to reconstitution of the complete HSP101 open reading frame and the simultaneous introduction of C-terminal HA tag (light blue) and an exported GFP reporter cassette that contains the KAHRP leader sequence. Pb101HA+2AmCh/K_L_GFP was created as for Pb101HA/K_L_GFP but with an additional 2A skip peptide (yellow) and mCherry (mCh) reporter inserted downstream of the HA tag. The predicted protein expressed in each transgenic parasite is shown. The 5′ and 3′ untranslated regions of HSP101 are indicated by blue arrows and blue rectangles, respectively. Oligonucleotides (labeled P1 to P4) used in diagnostic PCR analyses are indicated. SM, T. gondii DHFR-TS selectable marker cassette. (B) Diagnostic PCR with the indicated oligonucleotides confirms the expected integration events in the transgenic Pb101HA/K_L_GFP and Pb101HA+2AmCh/K_L_GFP parasite lines. (C) Representative Western blot analysis of parasite proteins extracted from transgenic P. berghei parasite lines using anti-HA antibodies (α-HA) (*n* = 3) show that cleavage at the 2A skip peptide is not complete in the Pb101HA+2AmCh/K_L_GFP lysate, with both cleaved (HSP101HA) and uncleaved versions of HSP101 (101HAmCh) present. EXP2 serves as a loading control. (D) Immunoprecipitation (IP) of mCherry in Pb101HA+2AmCh/K_L_GFP lysate using red fluorescence protein (RFP) TRAP agarose beads and probing with anti-HA or anti-EXP2 antibodies reveals that the uncleaved 101HAmCh protein interacts with HSP101HA protein and is incorporated into the PTEX complex (*n* = 5). GAPDH, which is not part of the PTEX complex, serves as a negative control. For loading, 20× elution fraction relative to supernatant has been used. (E) Sequential differential solubilization reveals that the uncleaved Pb101HA+2AmCh protein is not released into the hypotonic supernatant fraction (Hypo SN) but is instead present in the carbonate supernatant fraction (Carb SN) along with HSP101HA (*n* = 3).

To study HSP101 function and dissect how it contributes to protein export, two transgenic parasite lines were generated in the rodent malaria parasite Plasmodium berghei, termed Pb101HA/K_L_GFP and Pb101HA+2AmCh/K_L_GFP. To create these parasites, the endogenous *hsp101* locus was replaced with full-length *hsp101* fused at its 3′ end to a triple hemagglutinin (HA) and streptavidin epitope and incorporating a heterologous 3′ untranslated region (3′ UTR). Previous studies of Plasmodium falciparum and P. berghei have demonstrated that HSP101 can accommodate these tags at its C terminus without impacting parasite growth or protein export (21, 22). Utilizing the same construct, an exported reporter cassette was incorporated downstream of the *hsp101* locus of both lines. This reporter comprised the N-terminal leader of the knob-associated histidine-rich protein (KAHRP leader [K_L_]), including its hydrophobic signal sequence and PEXEL motif, conjugated to GFP and placed under the transcriptional control of the constitutive HSP70 promoter (48). The Pb101HA+2AmCh/K_L_GFP line also harbors an mCherry coding sequence, which is separated from the HSP101 coding sequence by the foot and mouth disease virus 2A skip peptide, to produce a polycistronic HSP101-HA-2A-mCherry mRNA (Fig. 1A and B).

We anticipated that the 2A skip peptide would produce separate HSP101-HA and mCherry proteins. However, Western blot analysis of HSP101 revealed that separation by the 2A peptide was inefficient in the Pb101HA+2AmCh/K_L_GFP line. Instead, two forms of HSP101 protein were generated, corresponding to HSP101-HA alone (Pb101HA) or a larger HSP101-HA+2A-mCherry protein (Pb101HA+2AmCh) (Fig. 1C). We have previously published a similar approach to generate parasite expressing PbEXP2-2A-FRT (herein renamed PbEXP2HA+2AmCh/K_L_GFP for consistency) in which EXP2 and mCherry were also generated from a polycistronic mRNA (for a schematic, see Fig. S1 in the supplemental material). In that case, cleavage of the 2A peptide was very efficient, giving rise to separate EXP2 and mCherry polypeptides (48).

10.1128/mBio.01106-19.1 FIG S1 Construct schematics. Schematic of PbEXP2 + 2AmCh/K_L_GFP (48) (A) and Pb101HA-mCh/K_L_GFP (B). Download FIG S1, EPS file, 1.8 MB. Copyright © 2019 Matthews et al. 2019 Matthews et al. This content is distributed under the terms of the Creative Commons Attribution 4.0 International license.

Since HSP101 oligomerizes into hexamers to associate with the PTEX complex, we examined whether conjugation of HSP101 with a bulky 28-kDa mCherry protein impaired the ability of HSP101 to be incorporated into PTEX. Immunoprecipitation of mCherry using red fluorescence protein (RFP)-TRAP resulted in the pulldown of Pb101HA+2AmCh together with Pb101HA. Importantly, Pb101HA+2AmCh also immunoprecipitated with EXP2. Together, this indicates that Pb101HA+2AmCh is incorporated into HSP101 hexamers and into the PTEX complex (Fig. 1D).

We then examined the solubility of Pb101HA+2AmCh using carbonate extraction assays (Fig. 1E). HSP101 is peripherally associated with the PVM and thus is normally released into the supernatant fraction only after treatment with carbonate. We found that Pb101HA+2AmCh has a solubility profile similar to that of Pb101HA, indicating that the addition of mCherry to Pb101HA did not change the solubility profile of the protein. This result is consistent with Pb101HA+2AmCh incorporating into the PTEX complex at the PVM, rather than being present as a soluble protein in the PV, in which case HSP101HA+2AmCh would be present in the hypotonic supernatant fraction.

### Pb101HA+2AmCh/K_L_GFP parasites fail to export the GFP reporter and show reduced parasite growth.

**FIG 2:**
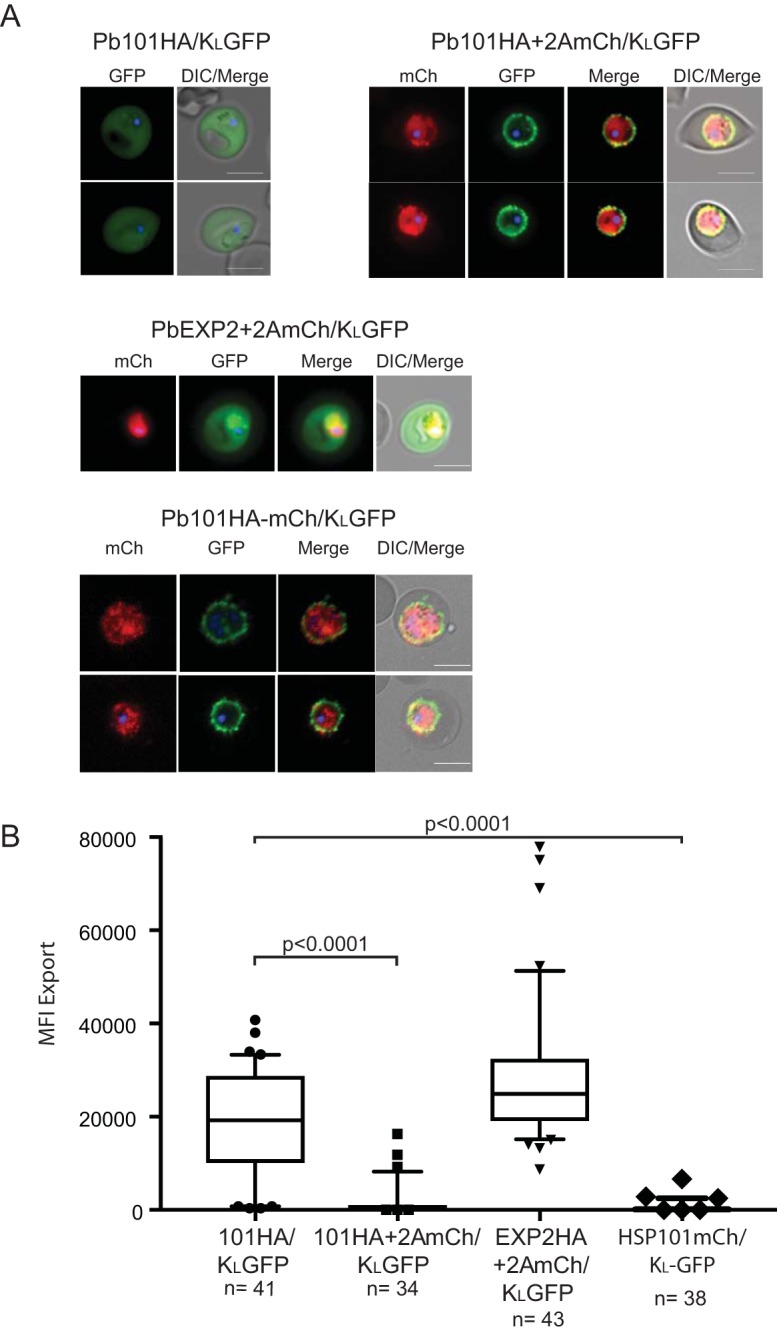
Live cell imaging of Pb101HA/K_L_GFP and Pb101HA+2AmCh/K_L_GFP. (A) Live cell fluorescence microscopy shows export of the K_L_GFP reporter into the cytosol of erythrocytes infected with Pb101HA/K_L_GFP, while the same reporter is largely prevented from being exported into erythrocytes infected with Pb101HA+2AmCh/K_L_GFP which instead localizes at the periphery of parasites which also express mCherry (mCh) (top panel). The control transgenic parasite line, PbEXPHA+2AmCh/K_L_GFP (middle panel), is capable of exporting the K_L_GFP reporter, while Pb101HA-mCh/K_L_GFP, which is analogous to PbHSP101HA+2AmCh/K_L_GFP but lacking the skip peptide (bottom panel), also fails to export GFP. All images were taken at ×1,000 magnification. DIC, differential interference contrast microscopy. Bars, 5 μm. (B) Quantitation of GFP export expressed as mean fluorescence intensity (MFI) ± the standard deviation (SD) in erythrocytes infected with the indicated parasite lines. The numbers of cells examined in three independent experiments are indicated. A *t* test was used to calculate statistical significance.

Strikingly, live cell imaging of the P. berghei transgenic parasite lines revealed the K_L_GFP reporter remained trapped at the PVM in the Pb101HA+2AmCh/K_L_GFP line (Fig. 2A, top panel). Only 11% of parasites were able to export GFP into the erythrocyte cytosol (Fig. 2B). In contrast, Pb101HA/K_L_GFP could efficiently export K_L_GFP (Fig. 2A, top panel, and Fig. 2B). We utilized PbEXP2HA+2AmCh/K_L_GFP parasites as a control to demonstrate that K_L_GFP can readily export GFP (Fig. 2A, middle panel, and Fig. 2B), consistent with what has been shown previously (48). We also created a P. berghei transgenic line, Pb101HA-mCherry/K_L_GFP line, which lacks the 2A skip peptide and thus endogenous HSP101 is tagged with mCherry (see Fig. S1 for a schematic). This line is analogous to the line previously described by Matz et al. but in addition contains the K_L_GFP reporter, as the ability to export GFP was not examined in that study (49). Similar to the Pb101HA+2AmCh/K_L_GFP line, Pb101HA-mCherry/K_L_GFP parasites failed to export GFP (Fig. 2A bottom panel, and Fig. 2B). Together, these experiments demonstrate that the addition of mCherry to the HSP101 hexamer is sufficient to block export of K_L_GFP through PTEX.

**FIG 3:**
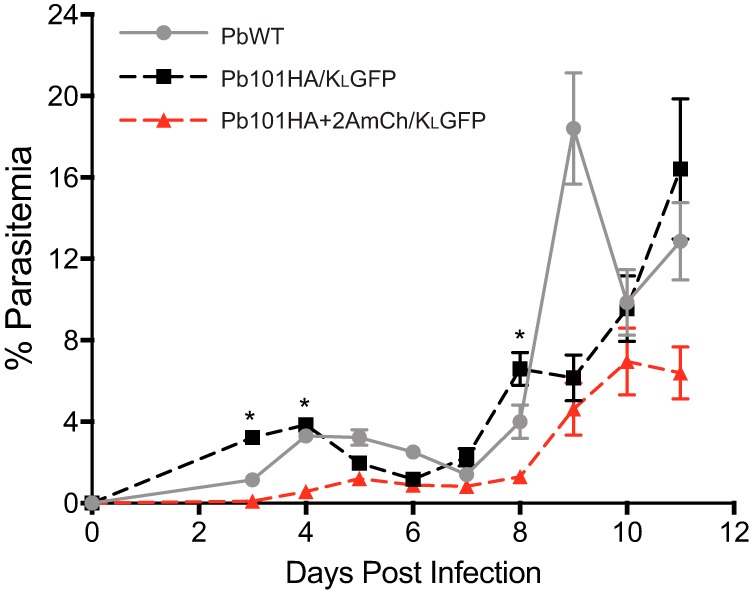
Comparative growth assay between wild-type P. berghei (PbWT), Pb101HA/K_L_GFP, and Pb101HA+2AmCh/K_L_GFP. Pb101HA+2AmCh/K_L_GFP grows significantly slower in BALB/c mice than PbHSP101HA/K_L_GFP does. Data were pooled from two separate experiments (*n* = 5 per experiment). Error bars represent standard errors of the means (SEM). A Student’s *t* test was used to calculate statistical significance, *, *P* < 0.05.

Comparative growth assays between wild-type (WT) P. berghei ANKA, Pb101HA/K_L_GFP, and Pb101HA+2AmCh/K_L_GFP in mice were also conducted. This revealed that Pb101HA+2AmCh/K_L_GFP took a significantly longer time to establish an infection compared to Pb101HA/K_L_GFP and also displayed delays in parasite growth (Fig. 3).

### Pb101HA+2AmCh/K_L_GFP can export native proteins.

**FIG 4:**
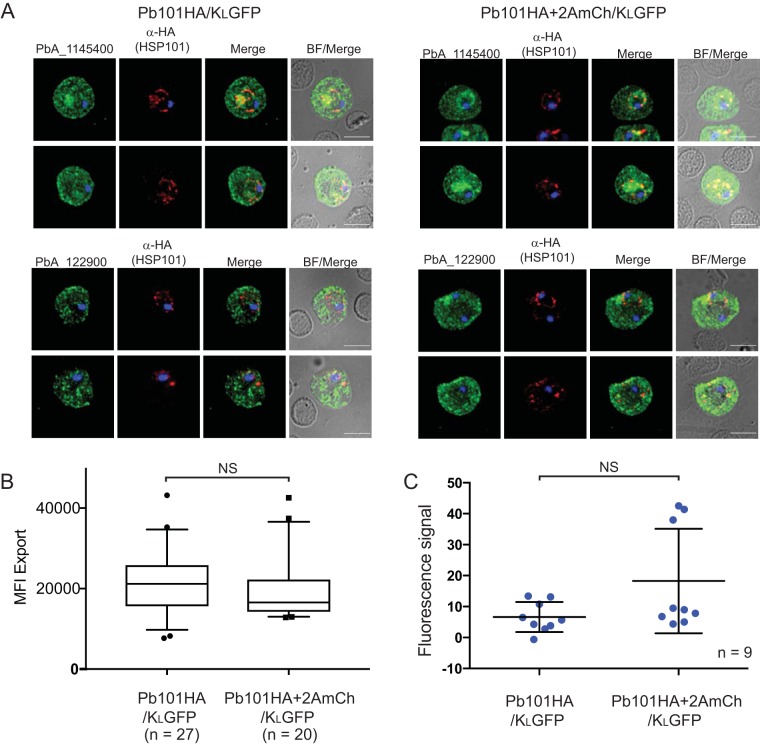
Native parasite protein export in Pb101HA/K_L_GFP and Pb101HA+2AmCh/K_L_GFP. (A) Representative IFA images of intraerythrocytic stages show that the native PEXEL proteins PbANKA_1145400 and PbANKA_1229000 are exported in both lines. Anti-HA antibodies detect HSP101HA located in the PV. While GFP cannot be exported in the Pb101HA+2AmCh/K_L_GFP line, native PbANKA_1145400 is exported to an extent similar to that of Pb101HA/K_L_GFP (left panel). BF, bright-field microscopy. (B) Quantitation of protein export of PbANKA_1145400 by measuring mean fluorescence intensity in the erythrocyte cytosol indicates there is no significant difference (*P* < 0.05) in export levels between the lines (*n* > 20 infected cells) (left panel). A Student’s *t* test was used to calculate statistical significance. Bars, 5 μm. (C) Labeling of parasite antigens on the surfaces of infected erythrocytes harvested between days 1 and 3 postinfection using semi-immune sera and quantitated by FACS. Representative graph shows no significant (NS) difference in fluorescence signal and thus protein export to the erythrocyte surface between the parasite lines (*n* = 9 biological repeats; error bars represent SD).

Experiments with P. falciparum and P. berghei have shown that inhibition of protein export via interference with PTEX function leads to parasite death (20, 22–29). Therefore, it was surprising that Pb101HA+2AmCh/K_L_GFP parasites, which fail to export GFP, were still viable in mice. Accordingly, we assessed the ability of two native P. berghei PEXEL-containing proteins that are members of the *Plasmodium* helical interspersed subtelomeric (PHIST) family to be exported via indirect immunofluorescence analysis (IFA). Unlike K_L_GFP, both of these PHIST proteins could be exported at similar levels in Pb101HA/K_L_GFP parasites and Pb101HA+2AmCh/K_L_GFP parasites (Fig. 4A). We quantified the level of export of PbANKA_1145400 in both these lines by measuring the mean fluorescence intensity (MFI) of signal in the erythrocyte and found that the parasite lines were not significantly different (Fig. 4B).

To determine whether the ability to export the two PHIST proteins is not limited to these two proteins, we next assessed the ability of P. berghei to export proteins to the erythrocyte surface using semi-immune P. berghei sera in flow cytometry assays as described previously (25). Similarly, we did not observe a significant decrease in protein export in the Pb101HA+2AmCh/K_L_GFP compared to Pb101HA/K_L_GFP (Fig. 4C). Indeed, protein export was enhanced in some experiments. Importantly, when the same parasite lines used in the fluorescence-activated cell sorting (FACS) experiments were analyzed for GFP localization, K_L_GFP was efficiently exported to the cytosol of erythrocytes infected with Pb101HA/K_L_GFP, whereas the K_L_GFP remained trapped at the parasite periphery in erythrocytes infected with Pb101HA+2AmCh/K_L_GFP (Fig. S2).

10.1128/mBio.01106-19.2 FIG S2 Live cell imaging of GFP in Pb101HA+2AmCh-infected erythrocytes. Live cell imaging of Pb101HA+2AmCh-infected erythrocytes shows that unlike native proteins that can be readily exported (Fig. 4), the K_L_GFP reporter protein remains trapped at the PVM. Download FIG S2, EPS file, 0.8 MB. Copyright © 2019 Matthews et al. 2019 Matthews et al. This content is distributed under the terms of the Creative Commons Attribution 4.0 International license.

Thus, while incorporation of Pb101HA+2AmCh into the HSP101 hexamer does not detectably decrease export of two PHIST proteins or global protein export, incorporation of the mCherry moiety does impact the ability of GFP to be exported. This indicates that the incorporation of mCherry into the HSP101 hexamer in the Pb101HA+2AmCh/K_L_GFP parasite has blocked the unfoldase activity of HSP101, but not the threading motor activity of the protein. That is, the threading function of HSP101 and its unfoldase activity have become uncoupled in this parasite line.

### Soluble reporter proteins are directly affected by the efficiency of HSP101 function.

GFP is a tightly folded protein that forms a beta-barrel structure. Previous studies have observed that highly overexpressed exported GFP reporters form a “necklace of beads” appearance within the PV in addition to being exported into the erythrocyte cytosol, suggesting inefficient translocation at the PVM (4, 8, 50). Similarly, P. falciparum parasites inefficiently export bovine pancreatic trypsin inhibitor (BPTI), which contains three disulfide bonds to form a tightly folded structure (20, 51). This indicates that unfolding tightly folded proteins may be a rate-limiting step in protein translocation across the PVM via PTEX. We hypothesized that incorporation of the Pb101HA+2AmCh into the HSP101 hexamer decreases the protein unfolding capacity of the complex, and in such cases would prevent translocation of very tightly folded proteins such as GFP.

**FIG 5:**
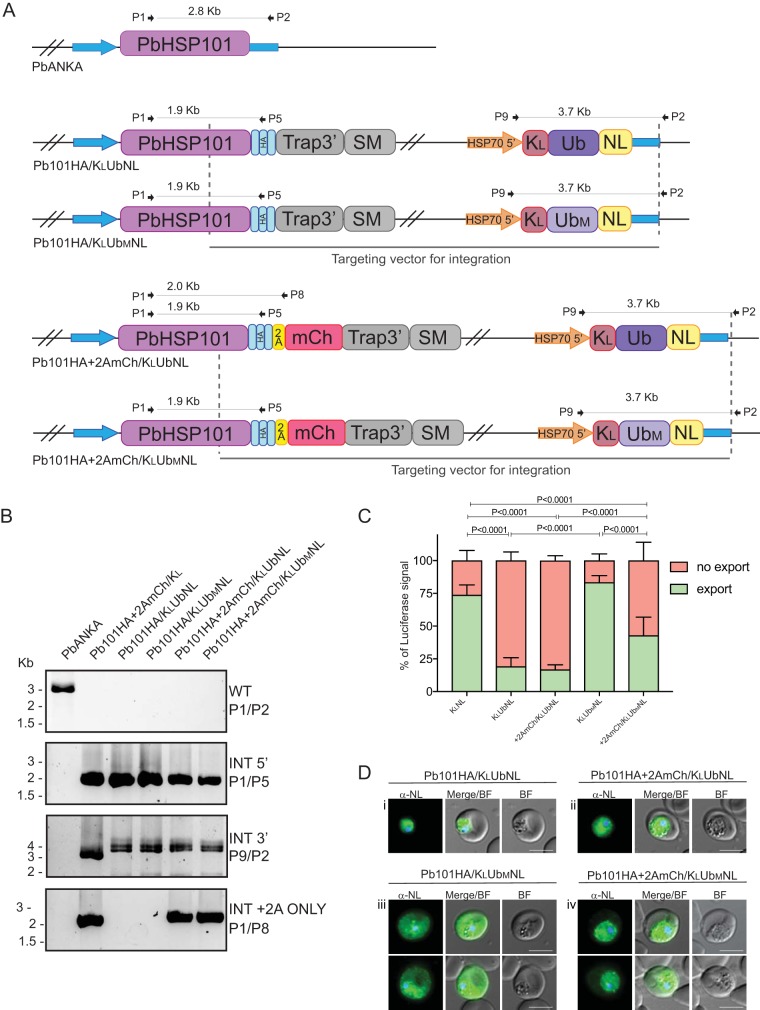
Generation and analysis of Pb101HA and Pb101HA+2AmCh parasite lines expressing derivatives of a ubiquitin reporter. (A) Schematic representations of transgenic parasite lines and the oligonucleotides used in diagnostic PCR analysis. SM, selectable marker; K_L_, KAHRP leader sequence; Ub, ubiquitin; Ub_M_, mutated ubiquitin; NL, nanoluciferase. (B) Diagnostic PCR with oligonucleotides as indicated confirms the successful generation of the transgenic parasite lines. INT, integrant. (C) Quantitation of nanoluciferase in the soluble (erythrocyte cytosol) and pellet (parasite plus PV space) fractions after treatment of infected erythrocytes with tetanolysin and centrifugation. Error bars represent SD. A Student’s *t* test was used to calculate statistical significance between groups. (D) Live cell imaging of infected erythrocytes used in panel C shows that both Pb101HA and Pb101HA+2AmCh parasite lines struggle to export the K_L_UbNL reporter. However, while mutation of ubiquitin leads to export of the K_L_Ub_M_NL reporter by Pb101HA, this reporter cannot be readily exported by Pb101-HA+2AmCh. Bars, 5 μm.

To test this hypothesis, we sought to determine whether other tightly folded proteins are blocked from being exported in Pb101HA+2AmCh parasites. To this end, we constructed an exported protein consisting of the P. falciparum KAHRP leader (K_L_) preceding the ubiquitin-60S ribosomal protein L40 (Ub), an 8.5-kDa protein conjugated to the nanoluciferase reporter (NLuc) (Fig. 5A). Ub is predicted to be a stably and very tightly folded protein (52). At the same time, we took advantage of known mutations of Ub which were predicted to be less tightly folded (Ub_M_), and conjugated this reporter to K_L_ and NLuc. In Ub_M_, isoleucine residues at positions 3 and 13 are replaced with alanine. These mutations decrease the conformational stability of ubiquitin without changing the overall folding pattern and have been previously used to assess the kinetics of protein translocation (52). Furthermore, the NLuc reporter (19.1 kDa) has been previously used to quantify protein export in P. falciparum (51, 53). All lines were validated for correct integration into the *hsp101* locus via PCR analysis (Fig. 5B).

We used tetanolysin to differentially permeabilize the erythrocyte membrane to quantify the level of NLuc activity in two fractions: the erythrocyte cytosol (exported fraction) and the PV compartment and parasite cytosol (nonexported fraction). Under these conditions, the KAHRP leader fused to the NLuc (K_L_NL) was efficiently exported into the erythrocyte cytosol (Fig. 5C). Quantification of the luciferase signal from the erythrocyte cytosol contributed to 73% of the total NLuc activity, meaning that only 27% of the NLuc remained in the PV and parasite fraction.

Unexpectedly, both the Pb101HA+2AmCh/K_L_UbNL and Pb101HA/K_L_UbNL parasite lines failed to efficiently export the K_L_UbNL (Fig. 5C). In both parasite lines, the NLuc activity from K_L_UbNL in the erythrocyte cytosol fraction contributed to less than 20% of the total luciferase activity, meaning that approximately 80% of NLuc remained in the PV and parasite fraction. This result was not statistically significant between the Pb101HA/K_L_UbNL and Pb101HA+2AmCh/K_L_UbNL parasites.

In contrast, we did observe a statistically significant difference between the export of K_L_Ub_M_NL between the Pb101HA and Pb101HA+2AmCh parasite lines. Parasites expressing Pb101HA exported K_L_Ub_M_NL as efficiently as K_L_NL, indicating that export of this reporter was not perceptibly impaired. However, in Pb101HA+2AmCh parasites, the amount of K_L_Ub_M_NL exported to the erythrocyte cytosol was significantly lower than in Pb101HA parasites, exporting 50% less total luciferase (Fig. 5C). This level of export indicates that the addition of mCherry to the HSP101 hexamer is sufficient to impair the export of K_L_Ub_M_NL across the PVM, albeit not entirely.

To visualize these quantitative findings, IFA was performed on Pb101HA+2AmCh/K_L_UbNL, Pb101HA+2AmCh/K_L_Ub_M_NL, Pb101HA/K_L_UbNL, and Pb101HA/K_L_Ub_M_NL lines using an anti-nanoluciferase antibody. In the parasite lines expressing wild-type Ub, we observed localization within the parasite cytosol, indicating that the presence of the K_L_ targeting motif, and specifically its recessed N-terminal signal peptide, was not sufficient to facilitate the trafficking of the tightly folded protein into the secretory system (Fig. 5D, panels i and ii). These results explain the low level of NLuc activity in the erythrocyte cytosol for both the Pb101HA and Pb101HA+2AmCh parasites as discussed above.

In contrast, parasites expressing Pb101HA, the K_L_Ub_M_NL reporter is clearly localized in the erythrocyte cytosol, in addition to the parasite cytosol and PV space (Fig. 5D, panel iii). This universal localization indicates that the mutations in Ub_M_NL are sufficient to destabilize the protein to allow secretion and export processes to occur, although some protein still remains in the parasite cytosol.

In Pb101HA+2AmCh parasites, the K_L_Ub_M_NL reporter is now only weakly localized in the erythrocyte cytosol. Instead, a “string of beads” pattern can be observed, indicating that the protein is trapped within the PV space as well as the parasite cytosol (Fig. 5D, panel iv). This localization indicates that the K_L_Ub_M_NL is being secreted into the PV, but the efficiency of export has been impaired by the addition of the mCherry tag to the HSP101 hexamer. The significantly diminished erythrocyte localization of K_L_Ub_M_NL is consistent with the decrease in NLuc activity in the exported fraction of Pb101HA+2AmCh.

### PEXEL proteins with TMDs are exported without unfolding by PTEX.

**FIG 6:**
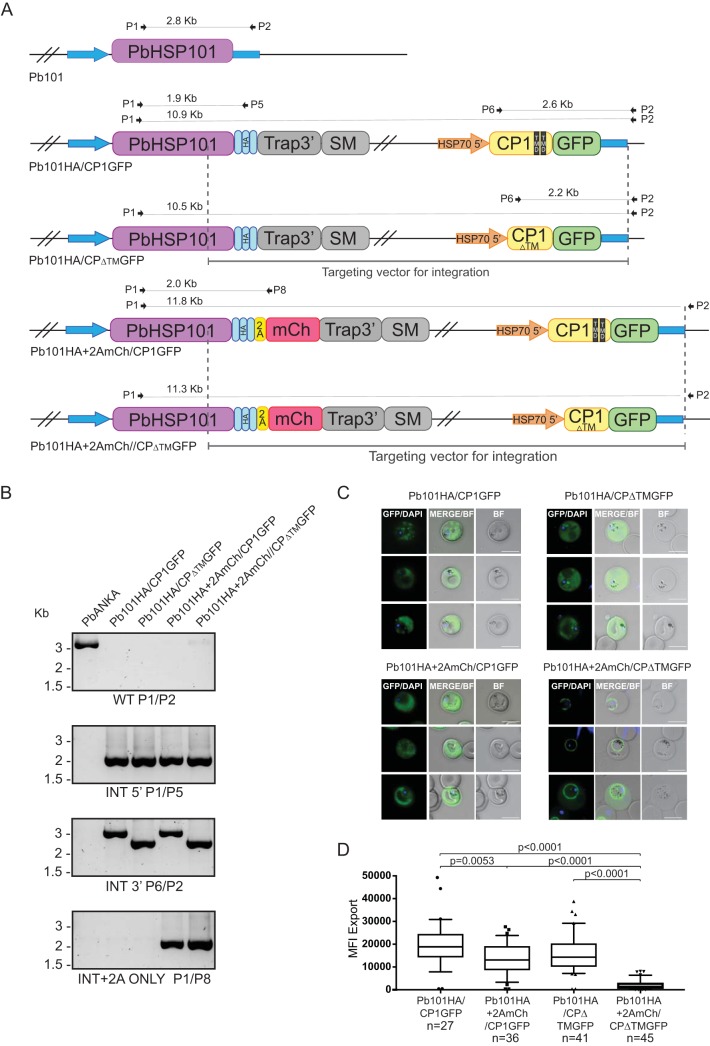
Generation and analysis of Pb101HA and Pb101HA+2AmCh parasite lines expressing derivatives of a CP1-GFP reporter. (A) Schematic representations of transgenic parasite lines and the oligonucleotides used in diagnostic PCR analysis. TMD, transmembrane domain; SM, selectable marker. (B) Diagnostic PCR with oligonucleotides as indicated confirms the successful generation of the transgenic parasite lines. PbANKA, P. berghei ANKA. (C) Live cell imaging of infected erythrocytes reveals Pb101HA+2AmCh can export CP1-GFP, which contains two TMDs, but when the TMDs are removed, the CP1_ΔTM_GFP reporter fails to be exported in the majority of cases. Bars, 5 μm. (D) Quantitation of GFP export as expressed as mean fluorescence intensity (MFI) ± SD in erythrocytes infected with the indicated parasite lines. The numbers of cells examined in three independent experiments are indicated. A *t* test was used to calculate statistical significance.

The exported reporters examined in the parasite lines above were all soluble proteins, which would be loaded into secretory vesicles from the ER and secreted into the PV by the fusion of the vesicle to the PPM, before engaging with HSP101 for translocation through PTEX. To determine whether exported PEXEL proteins with TMDs are secreted into the PV like soluble proteins or remain embedded in the PPM like PNEP proteins, we next created Pb101HA and Pb101 + 2AmCh lines expressing an exported PEXEL protein containing TMDs (Fig. 6A).

For this, we chose CP1 (PbANKA_124660), a protein which harbors an N-terminal signal sequence, PEXEL motif, and two TMDs toward its C terminus. We utilized both wild-type CP1 and CP1 mutants where the TMDs have been deleted (Fig. 6A). We have previously shown that full-length wild-type CP1 fused to GFP (CP1-GFP) is exported to membrane-like structures in the cytoplasm of infected erythrocytes and that deletion of both the TMDs from CP1 fused to GFP (CP1_ΔTM_GFP) does not decrease protein export but does prevent association with the membranous structures (50). We created Pb101HA and Pb101HA+2AmCh parasites expressing either CP1-GFP or CP1_ΔTM_GFP, and validated each parasite line for correct integration into the *hsp101* locus via PCR analysis (Fig. 6B).

We then measured the ability of GFP to be exported in live parasites (Fig. 6C and D). As expected, Pb101HA/CP1-GFP and Pb101HA/CP_ΔTM_GFP parasite lines could both efficiently export their respective reporters into the erythrocyte. Consistent with our previous study, removal of the TMDs altered the localization of GFP in the Pb101HA line from discrete punctate structures in the erythrocyte to an erythrocytic cytoplasmic localization (50).

In contrast, Pb101HA+2AmCh parasites could not export CP1_ΔTM_GFP (Fig. 6C and D). Instead, CP1_ΔTM_GFP, which is a soluble protein, accumulated at the parasite-host cell interface (Fig. 6C). This localization is consistent with the observation that parasites expressing Pb101HA+2AmCh are unable to export the soluble K_L_GFP reporter.

However, Pb101HA+2AmCh could export wild-type CP1-GFP efficiently (Fig. 6C and D). This counterintuitive result indicates that, unlike the soluble GFP reporters which are tightly folded in the PV, wild-type CP1-GFP protein is already in an unfolded/partially unfolded state prior to interacting with PTEX. This observation is consistent with CP1-GFP being embedded in the PPM and requiring extraction out of this membrane in order to be exported across the PVM by PTEX. The presence of TMDs in CP1 is sufficient to now enable CP1-GFP to be exported across the PVM.

## DISCUSSION

Although it has been nearly a decade since the original discovery of PTEX, the mechanism by which this complex facilitates the export of parasite proteins is only just beginning to be unravelled. This study focused on HSP101, one of the core components of PTEX, to provide insight into HSP101 function and how the complex is presented with and handles different types of cargo.

The use of the 2A skip peptide in molecular biology has provided an efficient method for dual protein expression under one promoter. In this study, however, placement of a 2A skip peptide between the P. berghei HSP101 coding sequence and a mCherry reporter led to an inefficient rate of 2A cleavage. Previous studies have demonstrated that the localized context of the 2A peptide can influence the cleavage efficiency of the skip peptide (54). It is unclear why self-cleavage of the 2A peptide was hindered in this case. Attenuation of this cleavage resulted in parasites that were able to express both Pb101HA and Pb101HA+2AmCh. Although we demonstrated that Pb101HA+2AmCh did integrate with Pb101HA into PTEX, we did not ascertain the stoichiometry of Pb101HA and Pb101HA+2AmCh. Nevertheless, our results clearly demonstrate that the addition of mCherry to HSP101 (either with or without the 2A skip peptide) diminished the unfoldase function of the HSP101 hexamer. By analyzing Pb101HA+2AmCh with various exported proteins, our study now provides direct evidence that HSP101 is an unfoldase but that compromised unfolding activity is sufficient for export of native proteins or proteins with TMDs.

In previous studies where HSP101 function was ablated by knocking down its expression or interfering with its assembly into the PTEX complex, protein export was completely blocked, resulting in parasites dying during the ring stages of the cell cycle (24, 25). In contrast, in the current study, we demonstrate that parasites expressing Pb101HA+2AmCh (and Pb101HA) remain viable and are able to complete the entire erythrocytic cell cycle. This viability is most likely due to the ability of the parasites to export native proteins at levels similar to those of parasites expressing only Pb101HA. Strikingly, however, we demonstrate that three different soluble GFP reporter proteins, trafficked by either the P. falciparum KAHRP leader or the P. berghei CP1 protein without TMDs, were unable to be exported by PbHSP101 + 2AmCh parasites and instead became trapped at the PVM. This was surprising, since GFP has been commonly used as a reporter in wild-type *Plasmodium* spp. to study sequences that direct protein export and PbEXP2 + 2AmCh parasites can also readily export GFP (48). Hence, the incorporation of HSP101 + 2AmCh into the HSP101 hexamer, while not affecting export of native cargo, affected its ability to export GFP, a beta-barrel protein that has a stable fold. This indicates that the unfoldase function of HSP101 may have been perturbed in this line, while the translocation function was not significantly disrupted. An alternate explanation is that tightly folded soluble cargo in the PbHSP101 + 2AmCh line could not gain access to the PTEX machinery at the PVM. For example, incorporation of mCherry into the HSP101 hexamer may have impeded HSP101 recognition of soluble protein cargo. However, this could be true only for tightly folded cargo, since soluble native proteins could still be exported in this line.

The fact that a fully functional HSP101 is required for export of tightly folded cargo such as GFP may explain why exoerythrocytic forms of the parasite, which do not express detectable levels of HSP101 (48, 49), fail to export mCherry or GFP reporters into their hepatocytic host cell, despite the presence of a PEXEL motif in these reporters (48, 55–57). It is also interesting to note that the related apicomplexan parasite, Toxoplasma gondii, which lacks a HSP101 homolog at the PVM can export native proteins but not highly folded proteins such as murine dihydrofolate reductase (mDHFR) and GFP into their host cell (58).

In addition to GFP, PbHSP101 + 2AmCh also failed to efficiently export the K_L_Ub_M_NL reporter, indicating that parasites expressing Pb101HA+2AmCh had a generalized reduction in protein unfolding activity that is not confined to unfolding GFP. However, K_L_Ub_M_NL was partially exported, albeit at significantly lower rates than in parasites expressing Pb101HA alone. This indicates that the unfolding activity of the HSP101 in Pb101HA+2AmCh parasites is not completely ablated and what activity remains is sufficient to unfold the Ub_M_ reporter. Indeed, approximately 11% of Pb101HA+2AmCh parasites was able to export K_L_GFP. This decreased, but not completely abolished, efficiency of HSP101 may account for the export of most native proteins observed in parasites expressing Pb101HA+2AmCh. A corollary prediction from this observation is that the majority of erythrocyte-stage exported proteins are unlikely to be as tightly and stably folded as the GFP beta-barrel, since we were unable to detect any significant decrease in export of proteins to the erythrocyte surface.

However, we cannot exclude the possibility that the unfolding function of the HSP101 hexamer is required to export an as yet undiscovered class of nonessential proteins that are tightly folded. An algorithmic screen of the total *Plasmodium* proteome for beta-barrel proteins (albeit those harboring transmembrane domains) that have a signal sequence revealed that not only are such proteins predicted to constitute a very small fraction (<0.2%), those that were identified are predicted to traffic to the chloroplast and mitochondrial membranes rather than be exported (59). Based on this observation, few exported TMD proteins are likely to be beta-barrel proteins. Consequently, consistent with our findings, a majority of exported proteins would not be affected by the decrease in unfolding activity caused by expression of Pb101HA+2AmCh.

Taken altogether, these results reveal that the HSP101 threading motor activity and unfoldase capacity of HSP101 have most likely become uncoupled in parasites expressing PbHSP101 + 2AmCh. The uncoupling of the ATPase and unfoldase activities in a ClpB protein has been observed previously in several studies that involved making defined alterations to the M domain of Escherichia coli ClpB (36, 37, 60). The M domain, which laterally protrudes from the ClpB ring, makes contact with the AAA-1 domain and is needed to solubilize aggregated proteins in E. coli. Unlike unfolded proteins, which can bind directly to the pore site of ClpB, aggregated proteins in E. coli require the DnaK/DnaJ/DnaE chaperone system to gain access to the pore site. A similar situation occurs in Saccharomyces cerevisiae, where the HSP70 chaperone system is required for its ClpB to disaggregate proteins. Mutational analysis of the M domain in E. coli revealed that not only does the M domain bind DnaK (37), it also serves as a molecular switch for ClpB activity (36, 37, 60). These elegant studies revealed ClpB mutants trapped in the repressed state are still able to thread soluble misfolded proteins and have ATPase activity similar to those of wild-type ClpB, but they do not have the unfolding power to process a tightly folded yellow fluorescence protein (YFP) or disaggregated proteins (36). Thus, we speculate that the addition of the mCherry tag to the HSP101 hexamer in Pb101HA+2AmCh parasites may be affecting the ability of the M domain to regulate the unfoldase function of HSP101, potentially by steric hindrance, without affecting the threading motor function of HSP101 because the ATP domains remain active.

It should be noted that ClpB proteins generally associate with DnaK (or HSP70) cochaperones to interact with substrates (61), but an equivalent cochaperone for HSP101 has not yet been identified in *Plasmodium* spp. Only *Laverania* species (including P. falciparum) possess a secreted HSP70 ortholog (HSP70-x) (34), but GFP can be exported in non-*Laverania* species (including P. berghei) that lack HSP70-x. In any case, HSP70-x is unlikely to be the cochaperone of HSP101 because it is exported into the erythrocyte cytosol (62). Whether HSP101 functions without a cochaperone remains to be confirmed. It is possible that the types of cargo HSP101 threads through its cavity do not require the activity of a cochaperone and that ER or PV chaperones are sufficient to prevent cargo from aggregating, keeping them in a soluble and competent state for translocation through PTEX.

Nevertheless, the parasite must still be able to regulate HSP101 activity in order to adapt to changing environmental conditions. The cryo-EM structure of PTEX demonstrated a possible interaction between the HSP101 M domain with a region of PTEX150 (30). The authors suggested that this may be one way in which the activities of HSP101 could be regulated. Thus, we predict that the incorporation of mCherry into the HSP101 hexamer may have interfered with this interaction with PTEX150, thereby dysregulating the unfolding activity of HSP101.

Finally, in contrast to the soluble reporters utilized in this study, we observed that CP1-GFP, which contains two TMDs, could be readily exported in parasites expressing Pb101HA+2AmCh. This indicates that it must have differed from the soluble GFP reporters in its fold state when presented to HSP101. One way in which this may have arisen is if the CP1-GFP protein was inserted into the PPM after vesicular trafficking from the ER. To traffic further, the reporter would have required extrusion out of the PPM, a process that would lead to the protein being in an unfolded form and thus able to be threaded through the HSP101 channel. While PNEPs have been shown to require extrusion out of the PPM to traffic further (13, 20), this is the first time that it has been shown that a similar mechanism may be in play for TMD-containing PEXEL proteins. How proteins are extracted out of the PPM is not clear, although the possibility that HSP101 plays a role in this process cannot be excluded.

In conclusion, we have constructed a variety of exported reporter proteins to demonstrate that the solubility and fold state of different types of exported proteins determines how they are translocated across the PVM by PTEX. We also demonstrate that HSP101 functions as an unfoldase but that only tightly folded proteins (and not native proteins or TM proteins) require the full unfolding capacity of HSP101 to be exported.

## MATERIALS AND METHODS

### Ethics statement.

All experiments involving rodents were performed in strict accordance with the recommendations of the Australian Government and National Health and Medical Research Council Australian code of practice for the care and use of animals for scientific purposes. The protocols were approved by the Animal Welfare Committee at Deakin University (approval no. G37/2013 and G08/2017).

### Plasmid construction.

For targeted integration of DNA constructs into the endogenous P. berghei *hsp101* locus, the C-terminal end of *hsp101* and the 3′ UTR were PCR amplified from P. berghei ANKA genomic DNA (gDNA) using gene-specific primers 461/462 and 459/460 (sequences provided in Table S1 in the supplemental material), respectively. The resulting fragments were digested with MluI/SacII and SacII/SphI and cloned into the corresponding sites of the previously described plasmid, pEXP2-2A-FRT (48). This plasmid contains the C-terminal end of *exp2* fused to sequences encoding a 2A skip peptide and mCherry reporter followed by a heterologous 3′ UTR as well as an exported reporter, KAHRP_L_-GFP, under the transcriptional control of the HSP70 promoter. The construction of the K_L_GFP expression cassette has been described previously (48). The final engineered construct was termed p101HA+2AmCh/K_L_GFP. A control construct, termed p101HA/K_L_GFP was made, in which the 2A-mCherry sequences were deleted. Both plasmids were sequenced and linearized with SacII prior to transfection.

10.1128/mBio.01106-19.3 TABLE S1 Oligonucleotides used in this study. Download Table S1, DOCX file, 0.02 MB. Copyright © 2019 Matthews et al. 2019 Matthews et al. This content is distributed under the terms of the Creative Commons Attribution 4.0 International license.

Additional plasmids were constructed using the parent plasmids described above with variations made to the reporter cassette. (i) To assess the trafficking of a tightly folded exported protein under the KAHRP leader sequence, the P. falciparum ubiquitin-60S ribosomal protein L40 (PF3D7_1365900) was amplified from P. falciparum gDNA using oligonucleotides DO617/DO618. Replacement of the isoleucine amino acids at positions 3 and 13 with alanine were made using the QuikChange II site-directed mutagenesis kit (Agilent) and oligonucleotides DO619/D0620. Fragments were digested with AvrII/AgeI and cloned into the corresponding sites of parent plasmids Pb101HA/K_L_GFP and Pb101HA+2AmCh/K_L_GFP. To enable biochemical measurement of the reporter wild-type and mutated ubiquitin proteins, the sequences encoding nanoluciferase (NLuc) were PCR amplified from pNL1 (Promega) using oligonucleotides DO749/DO750, and the resulting products were digested with AgeI*/*MluI and cloned in the corresponding sites of Pb101HA/K_L_GFP and Pb101HA+2AmCh/K_L_GFP in place of GFP. These four additional plasmids, termed Pb101HA/K_L_UbNL, Pb101HA/K_L_Ub_M_NL, Pb101HA+2AmCh/K_L_UbNL, and Pb101HA+2AmCh/K_L_Ub_M_NL were sequenced and linearized with SacII prior to transfection. (ii) To assess the trafficking of an exported protein containing a transmembrane domain (TMD), the TMD-positive CP1 protein was amplified from P. berghei gDNA using oligonucleotides DO595 and DO596. A truncated version of CP1, which was missing the C-terminal TMD was engineered using oligonucleotides DO595 and DO597. The CP1 and CP_ΔTM_ PCR products were digested with XhoI/AvrII and cloned into the corresponding sites of p101HA/K_L_GFP and p101HA+2AmCh/K_L_GFP. The four final constructs were designated Pb101HA/CP1-GFP, Pb101HA/CP1_ΔTM_GFP, Pb101HA+2AmCh/CP1-GFP, and Pb101HA+2AmCh/CP_ΔTM_GFP. All constructs were sequenced and linearized with SacII prior to transfection into P. berghei.

### Parasites and transfection.

The reference clone 15cy1 from the P. berghei ANKA strain was used to generate all P. berghei transgenic parasite lines. Transfection of parasites and selection of the transgenic parasites were performed as previously described (63). Briefly, Nycodenz-purified P. berghei schizonts were prepared for transfection, and DNA constructs were introduced using the Nucleofector electroporation device (Amaxa). The resulting DNA mixture was injected intravenously into 6- to 8-week-old BALB/c mice, and drug selection for genetically transformed parasites using pyrimethamine (0.07mg/ml) supplied in the drinking water was begun at day 1 posttransfection. The genotypes of all parasite lines were confirmed by PCR analysis of gDNA isolated from transgenic parasites using oligonucleotide combinations described in Table S1.

### Analysis of parasite growth.

To assess whether the growth and replication of transgenic Pb101HA/K_L_GFP and Pb101HA+2AmCh/K_L_GFP parasites were comparable, three groups of six BALB/c female mice at 6 weeks of age were infected with 1 × 10^6^ wild-type P. berghei ANKA, Pb101HA/K_L_GFP, and Pb101HA+2AmCh/K_L_GFP parasites. The parasitemia of each mouse was calculated from 3 days postinfection and thereafter on a daily basis by counting a minimum of 1,000 erythrocytes on Giemsa-stained blood smears. Mice were humanely culled when parasitemia exceeded 25%. Statistical analysis was performed using a Student’s *t* test.

### Live fluorescence microscopy.

For live fluorescence microscopy*,* whole infected blood was collected daily by tail snip followed by 5-min incubation with 4′,6′-diamidino-2-phenylindole (DAPI) (5 mg/ml), the cells were briefly washed in 1× mouse tonicity phosphate-buffered saline (MT-PBS). Erythrocytes were placed onto glass slides with a coverslip and imaged using a Nikon Ti2 microscope. Mean fluorescence intensity (MFI) data were collected using ImageJ version 2 (http://imagej.net) using the “measure” function whereby the total fluorescence measured within the parasite was subtracted from the total fluorescence signal of whole infected cells. MFI data were graphed using GraphPad Prism version 7.

### Indirect IFA.

For immunofluorescence microscopy, infected erythrocytes were fixed with ice-cold 90% acetone/10% methanol for 2 min, prior to blocking in PBS containing 1% bovine serum albumin (BSA). For nanoluciferase (nanoluc) indirect immunofluorescence analysis (IFA), fixation was instead performed with 0.025%/4% glutaraldehyde/paraformaldehyde, followed by 0.25% Triton X-100 permeabilization. All antibody incubations were performed in PBS containing 0.5% BSA for 1 h at room temperature. Primary antibodies for P. berghei blood stages were used at the following concentrations: rabbit anti-PbA_114540 (64), rabbit anti-PbA_122900 (64), rabbit anti-nanoluc (65), and polyclonal rabbit anti-EXP2 all at 1:1,000 (31), and rat anti-HA and mouse polyclonal anti-P. berghei at 1:500 (25). After three washes with PBS, the appropriate Alexa Fluor 488/568-conjugated secondary antibodies were used at 1:5,000 (Molecular Probes) and mounted on glass slides using ProLong Antifade with DAPI (Thermo Fisher). Images were acquired with an Olympus IX70, Leica TCS SP2 or Nikon Ti2 microscope, and images were processed using ImageJ version 2 (http://imagej.net) or Adobe CS6 Photoshop.

### Measure of protein export by FACS analysis.

For analysis of P. berghei antigens on the infected erythrocyte surface using fluorescence-activated cell sorting (FACS), P. berghei*-*infected erythrocytes were labeled with either P. berghei semi-immune or nonimmune (prebleed) sera as previously described (25). Samples were analyzed using a FACS Canto II machine (BD Biosciences). Fluorescence signal was quantitated by subtracting the prebleed fluorescence signal from the immune serum fluorescence signal.

### Protein analysis and immunoprecipitations.

Infected rodent erythrocytes (depleted of leucocytes by CF11 treatment [Whatman]) were lysed using 0.03% saponin containing 1× complete protease inhibitors (Roche) by incubating for 15 min on ice, followed by centrifugation at 20,000 × *g* for 5 min. After three washes with ice-cold mouse tonicity (MT) PBS containing protease inhibitors, the parasite pellet was resuspended in 1× reducing sample buffer. For immunoblots, P. berghei parasite lysates were separated on a 4 to 12% gradient SDS-polyacrylamide gel (Invitrogen) and transferred onto a 0.45-μm nitrocellulose membrane (Millipore) using 1× Trans-Blot Turbo transfer buffer (Bio-Rad) with an iBlot transfer device (Bio-Rad). Immunoreactions were performed using antibodies in PBS containing 3% BSA, and detection was performed using Clarity Max Western ECL Blotting substrate (Bio-Rad).

For immunoprecipitations (IP), P. berghei-infected red blood cells (RBCs) depleted of leucocytes were lysed with 0.03% saponin. Pelleted parasite material was lysed with 1% Triton X-100 in PBS containing complete protease inhibitors (Roche). After a 1-h incubation on ice, the material was centrifuged at 21,000 × *g* for 10 min at 4°C. The supernatant was affinity purified with RFP-Trap A agarose beads (ChromoTek) per the manufacturer’s instruction for 4 h at 4°C. Unbound material was collected, and the beads were then washed four times with 1 ml lysis buffer. Bound proteins were eluted with 50 μl of 2× reducing sample buffer and separated by SDS-PAGE. For immunodetection, the following primary antibodies were used: monoclonal mouse anti-HA (12CA5) (1:1,000) (Roche), polyclonal rabbit anti-EXP2 (1:1,000), and polyclonal rabbit anti-glyceraldehyde-3-phosphate dehydrogenase (anti-GAPDH) (1:1,000). Secondary antibodies were horseradish peroxidase-conjugated goat anti-mouse and goat anti-rabbit antibodies (1:4,000; Thermo Fisher). Experiments were repeated on five independent occasions, with representative data shown.

### Nano-luciferase export assay.

Luciferase export assays were performed on P. berghei erythrocytes infected with parasites harboring the NLuc expression plasmids Pb101HA/K_L_UbNL, Pb101HA/K_L_Ub_M_NL, Pb101HA+2AmCh/K_L_UbNL, and Pb101HA+2AmCh/K_L_Ub_M_NL and control plasmid Pb101HA/K_L_NL. When parasitemias of the mice reached between 2 and 5%, 10 μl of peripheral blood was collected via tail snip and suspended in 15 μl of 1×MT/PBS. To puncture the erythrocyte membrane, tetanolysin (Sigma) was added to a final concentration of 1.6 ng/μl. Cells were incubated for 10 min at room temperature under agitation at 600 rpm, after which they were centrifuged at 3,000 rpm to separate the host cytosolic (supernatant [SN]) and erythrocyte membrane and parasite pellet fractions. The pellets were then resuspended and completely lysed in a total volume of 50 μl to match the total SN volume to maintain equal ratios. To measure the Nanoluc signal, equal volumes of SN and pellet fractions were added to 100 μl of diluted NanoGlo (Promega) per the manufacturer’s instructions. The relative light units were then measured using a Berthold Lumat LB 9507 luminometer (Berthold Technologies, Bad Wildbad, Germany) where the exported and nonexported ratios were calculated as a percentage of the total NLuc expressed. Experiments were repeated on six independent occasions, and two technical replicates were completed per biological replicate.

### Differential extraction.

To differentiate cytosolic, peripheral, and integral membrane parasite proteins, infected erythrocytes at ring stage were lysed with 0.03% (wt/vol) saponin in PBS supplemented with protease inhibitor cocktail (Roche). The parasite material was divided equally between three tubes, and the parasite material in each tube was resuspended in either hypotonic lysis buffer (1 mM HEPES [pH 7.4]) or carbonate extraction buffer (0.1 M Na_2_CO_3_ [pH 11.5]) on ice or membrane extraction buffer (1% [wt/vol] Triton X-100 in PBS) at room temperature for 30 min. All samples were then centrifuged at 100,000 × *g* for 30 min at 4°C. Reducing sample buffer (1× final concentration) was added to the supernatant and pellet fractions, and samples were separated on SDS-polyacrylamide gels prior to analysis by Western blotting. Experiments were repeated on three independent occasions.

### Statistical analysis.

Graphs were generated, and data were analyzed using GraphPad Prism 7.0b software (MacKiev). Statistical analysis was performed using a Student’s *t* test. A *P* value of <0.05 was considered significant.
