# Supplementary material for: Uncoupling the Threading and Unfoldase Actions of Plasmodium HSP101 Reveals Differences in Export between Soluble and Insoluble Proteins
Source: mBio. 2019 Jun 4;10(3):e01106-19. doi: 10.1128/mBio.01106-19 (PMC6550532; doi:10.1128/mBio.01106-19)
Supplement: TABLE S1 [file mBio.01106-19-s0001.docx]

**Table S1:** Oligonucleotides used in this study

| **Primer ID/Name** | **Application** | **Sequence (5´-3´)** |
| --- | --- | --- |
| P1 | HSP101 genotyping | gagccgcgGAATAAGATAATATTATTTGTCGATGAA |
| P2 | HSP101 genotyping | GTACCAAACCGCAAATATCTAATCC |
| P3: | TRAP3’ UTR Rev | CAATTTAAAgacaaacgagtttatgag |
| P4 | GFP For | GGGATTACACATGGCATGGATG |
| P5 | HA Reverse (NotI) | gtagcggccgcttaGGCATAATCTGGAACATCGTACGGATA |
| P6 | CP1 F | caccctcgagATGAGAGTCAGTATTTTAAAATATGTTC |
| P8 | mCherry Rev | GCCATGTTATCCTCCTCGC |
| P9 | HSP70 5' For | caccaagcttGTAATATTTTGTTGGTGAGCTTA |
| DO459 | HSP101 3' UTR | cagacgcgtTAGGAAAAAACATTCCCGTATAGAAT |
| DO460 | HSP101 3' UTR | ttaccgcggAATTTTATATGATTTTTATTTATCGAGCAAA |
| DO461 | HSP101 5' UTR | ccgcggATTTAAGAGATTCCGGTATG |
| DO462 | HSP101 3' UTR | ttagcatgcTGACAATGAAAGGTTAATAACAATGTT |
| DO595 | CP1 | caccctcgagATGAGAGTCAGTATTTTAAAATATGTTC |
| DO596 | CP1 | tatcctaggTTTTGGTATTTTTTTTAATTTAAAGTTACATTTA |
| DO597 | CP1_ΔTM_ | atcctaggTGTTTTACTTCCTTTATGCTTCTTTATAT |
| DO617 | Pf3D7_1365900 | caccctaggATGCAAATTTTTGTAAAAACATTAAC |
| DO618 | Pf3D7_1365900 (ubiquitin) | tcgacgcgtTTAaccggtTCCTCCTCTTAATCTTAAAACc |
| DO619 | To mutagenize ubiquitin | CATTAACTGGAAAAACAggaACCCTTGATGTTGAGCC |
| DO620 | To mutagenize ubiquitin | caccctaggATGCAAggaTTTGTAAAAACATTAACTGGAAAAACAgga |
| DO749 | Nanoluc | TATcctaggTAAaccggtATGGTCTTCACACTCGAAGAT |
| DO750 | Nanoluc | TTAcgcgtTTACGCCAGAATGCGttcgca |

**Supplementary Figure Legends**

**FigS1. Construct schematics**

Schematic of (A) PbEXP2+2AmCh/K_L_GFP (Kalanon et al., 2016) and (B) Pb101HA-mCh/K_L_GFP.

**FigS2. Live cell imaging of GFP in Pb101HA+2AmCh infected erythrocytes**

Live cell imaging of Pb101HA+2AmCh infected erythrocytes shows that unlike native proteins that can be readily exported (Fig. 4) the K_L_GFP reporter protein remains trapped at the PVM.
